# Unlocking the genetic secrets of Dorper sheep: insights into wool shedding and hair follicle development

**DOI:** 10.3389/fvets.2024.1489379

**Published:** 2024-12-11

**Authors:** Xiaochun Yuan, Ke Meng, Yayan Wang, Yifan Wang, Cuili Pan, Haoran Sun, Jankui Wang, Xinhai Li

**Affiliations:** ^1^College of Animal Science and Technology, Ningxia University, Yinchuan, China; ^2^Beijing Key Laboratory of Animal Genetic Improvement, China Agricultural University, Beijing, China

**Keywords:** Dorper sheep, RNA-Seq, hair follicles, transcript, WGCNA, alternative splicing events

## Abstract

Dorper sheep is popular among farming enterprises with strong adaptability, disease resistance, and roughage tolerance, and an unique characteristic of natural shedding of wool. In a large number of observations on experimental sheep farms, it was found that the wool of some sheep still had not shed after May, thus manual shearing was required. Therefore, understanding the molecular mechanisms of normal hair follicles (HFs) development is crucial to revealing the improvement of sheep wool-related traits and mammalian skin-related traits. In this study, transcriptome analysis was performed on skin tissues of adult Dorper ewes in the shedding (S) and non-shedding (N) groups in September 2019, January 2020, and March 2020, respectively. The results identified 3,278 differentially expressed transcripts (DETs) in the three comparison groups within the S group, 720 DETs in the three comparison groups within the N group, and 1,342 DETs in the three comparison groups between the S-*vs*-N groups. Time-series expression analysis revealed 2 unique expression patterns in HF development, namely, elevated expression in the anagen phase (A pattern) and the telogen phase (T pattern). DETs with stage-specific expression had a significant presence in processes related to the hair cycle and skin development, and several classic signaling pathways involved in sheep HF development, such as Rap1, estrogen, PI3K-Akt, and MAPK, were detected. Combined analysis of DETs, time-series expression data, and weighted gene co-expression network analysis identified core genes and their transcripts influencing HF development, such as *DBI*, *FZD3*, *KRT17*, *ZDHHC21*, *TMEM79*, and *HOXC13*. Additionally, alternative splicing analysis predicted that the isoforms XM_004004383.4 and XM_012125926.3 of *ZDHHC21* might play a crucial role in sheep HF development. This study is a valuable resource for explaining the morphology of normal growth and development of sheep HFs and the genetic foundation of mammalian skin-related traits. It also offers potential insights into factors influencing human hair advancement.

## Graphical abstract

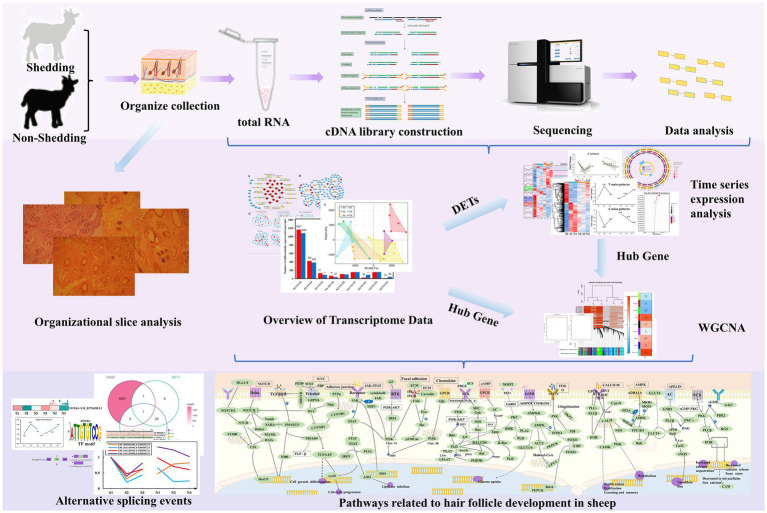

## Introduction

The hair follicles (HFs) of sheep constitute a “micro-organ” of the skin (1, 2). They are essential for sebum and sweat secretion, temperature regulation, homeostasis maintenance, and the regeneration and repair of skin stem cells (2). Additionally, HFs are an important source of tissues, which promote wool growth and determine the periodic growth characteristics of hair (3). Sheep HFs comprise primary hair follicles (PHFs) with medullated hair and secondary hair follicles (SHFs) without medullated hair. Sheep wool is derived from the SHF structures in the skin (4, 5). The HFs of sheep and wool growth and development follow a cyclic pattern of anagen, catagen, and telogen phases, which are influenced mainly by external environmental factors and specific genetic factors (6). The Wnt signaling pathway plays a crucial role in epidermal development, HF morpho-genesis, and regeneration (7). Gab1, which is downstream of receptor tyrosine kinase and upstream of Shp2 and Mapk, is involved in the regulation of the HF cycle and self-renewal of follicular stem cells (8). In normal skin, BMP controls epidermal homeostasis, HF growth, and melanogenesis (9). The *APC* (exon skipping), *POFUT1* (intron retention), and *TGFBR3* (cassette exon) genes, which are associated with HF development, are regulated by selective splicing (10). Transcription factors such as *KLF4*, *LEF1*, *HOXC13*, *RBPJ*, *VDR*, *RARA*, and *STAT3* are specifically expressed in HF morphogenesis (11). Moreover, wool growth is a seasonal and cyclic phenomenon in animals that is controlled by photoperiod, inhibitory signaling, and the endocrine system (12, 13).

Interactions of multiple signaling pathways and specific genetic factors regulate HF growth and development cycle. The Wnt and Hippo signaling pathways promote Cashmere goat HF growth, whereas the Rap1, PI3K-Akt, NF-kappaB, and cAMP signaling pathways are crucial in the catagen and telogen phases. The PI3K-Akt signaling pathway and extracellular matrix (ECM) receptor interactions play important roles in transitioning from the telogen to the anagen phase, serving as candidate biomarkers for the regeneration process (14). *FGF5*, *FGFRL1*, and *RRAS* genes affect HF development through the MAPK signaling pathway in the Inner Mongolian velvet goat (5). Vimentin regulates the growth cycle of Cashmere goat HFs by affecting the outer root sheath (15). A comparison of molecular signals that initiate HF development on days 45, 55, and 65 in the embryos in Cashmere goats revealed significant differences in the Wnt, TGF-*β*, FGF, Hedgehog, and NOTCH signaling pathways from E55 to E65. *FOXN1*, *GATA3*, and *DLX3* may have consistent effects on HF development (10). The Notch signaling pathway plays a crucial role in HF differentiation and maturation, with regulatory factors such as *FOXN1*, *HOXC3*, *PRR13*, and *LHX15* potentially having consistent regulatory roles in Cashmere goat HF development (16). *KRT25*, *KRT27*, *KRT19*, *KRT10*, *KRT77*, *KRT1*, *KRT24*, *KRT14*, and *KRT4* are considered to be markers of the HF cycle (17). Additionally, the genes *LAMA5*, *WNT10A*, *KRT25*, *ZDHHC21*, *FZD1*, *LRP4*, *TGFβ2*, *TMEM79*, *SOX10*, *ITGB4*, and *KRT14* were significantly enriched in epidermal differentiation and development, hair follicle development, and hair follicle morphogenesis, and they were expressed specifically in the wool follicles of sheep at different developmental stages (18). In general, research on hair follicle development in cashmere goats has been widely reported, but research on wool follicles in sheep is relatively rare. In addition, the automatic shedding feature of Dorper sheep saves a certain amount of production costs for breeding companies, while research on some Dorper sheep that do not automatically shed their hair is even less common.

Weighted Correlation Network Analysis (WGCNA) allows for the joint analysis of high-throughput and phenotypic data, categorizing genes with similar expression profiles in modules and defining key modules and genes (19, 20). WGCNA has been used to identify key genes such as *WNT10A*, *KRT14*, *WNT11*, *LEF1*, *WNT5A*, *KRT1*, and *KRT6*, which are associated with the development of the HF cycle in Inner Mongolian velvet goats. Among these, *WNT10A* is a crucial gene that regulates the development of HFs (18, 21, 22). ECM-receptor interactions, adhesion patches, the PI3K-Akt signaling pathway, and estrogen signaling pathways are closely related to the cyclic development of HFs. Genes such as *COL1A1*, *C1QTNF6*, and *COL1A2* play a role in increasing cashmere production (23). In addition, alternative splicing, as an important regulatory modality, has an intrinsic transcriptional regulatory mechanism that results in polymorphisms in transcript and protein structure and function. *FGF5* signaling is expressed at a much higher level in the final phase of the anagen phase than in the telogen phase, inducing a transition to the catagen phase. In contrast, *FGF5* signaling plays an inhibitory role in the anagen-to-catagen transition in the dermal cells of Cashmere goats (24, 25).

Numerous studies have demonstrated that wool follicle development is regulated by various pathways and specific genes throughout the growth cycle. However, most studies have focused on wool follicles and wool growth in Cashmere goats, with few studies addressing the entire cycle of wool follicle development and growth in sheep. Moreover, there are relatively few reports of Dorper sheep not shedding automatically. Additionally, regulation of the expression of single-gene multiple transcripts generated from mRNA precursors (pre-mRNAs) through different splicing modes during the cyclic changes in wool follicle development and growth in sheep have not been extensively studied, and the regulatory mechanisms that are involved remain largely unexplored. Furthermore, research on the impact of alternative splicing events on HF development in sheep is limited. In this study, transcript expression datasets were analyzed to identify key transcripts, pathways, and variable splicing events affecting the regulatory mechanisms of normal hair follicle development and growth cycles in sheep. Our study aims to provide a theoretical foundation to elucidate the regulatory mechanisms underlying the cyclic wool follicle growth in sheep.

## Materials and methods

### Animals and sample collection

All adult Dorper ewes used in this study were obtained from Zhong mu Yilin Livestock Co. Ltd. (Yinchuan, China). Sustained observations at the experimental sheep farm revealed that Dorper sheep exhibited a trait-separation phenomenon between shedding and non-shedding wool. By May, some sheep continued to retain their wool and required manual shearing. Consequently, Dorper adult ewes with consistent feeding and management conditions, good body condition, and similar age (2 years) were selected for this study. Ewes were categorized into a shedding group (S) and a non-shedding group (N) to investigate the cyclic development of HFs. Initially, 10 Dorper sheep were selected from the S group and the N group as the experimental model. Five Dorper sheep from the group S and three from the group N with the best phenotype were finally selected for subsequent analysis. The samples were collected in September 2019, January 2020, and March 2020. Skin tissues were collected from the same batch of sheep in group S (5 sheep) and group N (3 sheep) at each time point. Samples were collected from the posterior edge of the last rib at the junction of the rib and the midline of the body (Sample size: 2cm^2^). For sampling, the wool is first sheared as far as possible. Next, the sampling site was disinfected and injected with an anesthetic drug. Then, after the skin tissue was cut off using scissors, the sheep wounds were sprayed with iodophor. Finally, anti-inflammatory drugs were applied to the wounds, and the wounds were sutured. We have done our best to minimize the suffering of the sheep. Tissue samples were rinsed with phosphate-buffered saline, immediately transferred to RNAase-free cryo-preservation tubes, and preserved in liquid nitrogen. The stages of Group S were labeled as S1, S2, and S3, whereas those of Group N were labeled as N1, N2, and N3 according to the sampling time. Additionally, skin tissues were collected for paraffin sectioning.

### Histological staining

After immersing sheep skin samples in 4% paraformaldehyde for 24 h for fixation, the samples were embedded and rinsed for 30 min with running water to eliminate the fixative and then dehydrated in gradient ethanol solutions. The tissue was embedded in paraffin using a JB-P5 machine. Paraffin blocks were sectioned along the horizontal axis using an RM2016 microtome (Germany) to yield Sections approximately 5 μm thick. The sections were photographed at 40× magnification using an OLYMPUS cellSens (BX53) microscope. PHF and SHF diameters were measured using ImageJ software.^1^ Data analysis was conducted using one-way ANOVA with the Agricola package (v.1.3–7) for R studio software.

### Total RNA extraction and transcriptome sequencing

Total RNA was isolated from 24 skin samples using TRIzol reagent (Invitrogen, United States). RNA purity, concentration, and integrity of each sample were checked with a Nanodrop 2000 and an Agilent 2,100 Bioanalyzer with RIN ≥ 8.4, and 1.8 < optical den-sity260/280 < 2.0. An RNA-seq library was constructed using a TruSeq™ RNA sample preparation kit (Illumina; San Diego, CA) following the manufacturer’s instructions. First, ribosomal RNA was removed to maximize the retention of all coding RNAs. The resulting RNA was then randomly fragmented into short pieces of approximately 300 bp. Subsequently, these RNA fragments served as templates for synthesizing the first strand of cDNA using six-base random hexamers. Next, the second strand of cDNA was synthesized by adding buffer, dNTPs (with dUTP instead of dTTP), RNase H, and DNA polymerase I, followed by purification using a QiaQuick polymerase chain reaction (PCR) kit and elution with EB buffer for terminal repair, the addition of base A, and sequencing adapter. Degradation of the second chain was achieved using the enzyme uracil-N-glycosylase. Lastly, PCR amplification and fragment size selection were performed using agarose gel electrophoresis. The constructed sequencing library was then sequenced on the Illumina HiSeq™ 4,000 platform with 150 bp paired-end reads. Each sample generates approximately 6 Gb of sequencing data.

### Data quality control, comparison, assembly, and expression calculation

The following analysis was done based on MobaXterm (v.23.1). Quality filtering of raw data was performed using FastQC (v.0.11.9) software to remove reads containing adapter sequences, reads with >10% N content, duplicate reads, and low-quality reads (where bases with a quality value Q ≤ 20 made up more than 50% of the total reads), to obtain clean reads. HISAT2 (v.2.2.1)^2^ (26) was used to align the reads to the sheep reference genome (Oar_rambouillet_v1.0), and StringTie (v.2.2.0) (27) software was used to assemble the transcripts and calculate the transcript expression in each sample. The expression level is displayed as raw reads count and FPKM. The raw reads count indicates the number of reads contained in the transcript, but due to the influence of sequencing amount and transcript length, the raw reads count is not conducive to the comparison of differential transcripts between samples. To ensure the accuracy of subsequent analysis, we first corrected the sequencing depth, then corrected the length of the transcript, and obtained the FPKM value of the transcript before conducting subsequent analysis.

### Bioinformatics analysis

#### Sample relationship analysis

The following analysis was performed based on R studio. In order to understand the repeatability between samples and assist in excluding abnormal samples. The gmodels package (v.2.19.1) was used to perform principal component analysis (PCA). The corrplot package (v.0.92) was used to perform a Pearson correlation analysis on all samples.

#### Identification of differential transcripts

Normalization, *p*-value calculation, and multiple hypothesis testing corrections (false discovery rate, FDR) were conducted using the DESeq2 (v.1.42.0) package on each sample’s read count (28). Differentially expressed transcripts (DETs) were identified using |log2FC| ≥ 1 and P-adjust <0.05 as the criteria. Quantitative relationships of DETs among groups were plotted using GraphPad (v.8.0.1), and the intersecting genes among the DETs were visualized using the UpSetR package (v.1.4.0).

#### Enrichment function analysis and network visualization

Functional annotation and enrichment of all transcript from sequencing were performed using Gene Ontology (GO) and Kyoto Encyclopedia of Gene and Genomes (KEGG) databases. Gene network interactions were visualized using Cytoscape (v3.9.1) (29).

#### Time series expression analysis

The pheatmap package (v.1.0.12) was used to visualize the clustering among samples, and the STEM software^3^ was used to classify the expression patterns of DETs and identify the expression patterns and key transcripts related to HF development.

#### Weighted correlation network analysis

Co-expression networks were constructed using the WGCNA (v.1.72–5) package. Use the pickSoftThreshold function to select the appropriate soft threshold power *β* value to calculate the network structure. Merging modules with 75% feature transcript similarity using a dynamic tree-cutting technique to ascertain the final module count. Transcript with |MM| ≥ 0.75 in key modules were correlated with hub transcript (Demonstrated as a gene in the main text) related to HF development in sheep to pinpoint the hub transcript (Demonstrated as a gene in the main text) and their regulatory relationships with HF development pathways. In addition, sequence motif enrichment in the promoters of transcripts specifically expressed during the stages related to HF development was analyzed using MEME (v. 5.5.5).

#### Alternative splicing analysis

Alternative splicing is a crucial gene regulatory mechanism in eukaryotes. RMATS (30) was used to determine 5 variable splicing events, namely, exon skip (ES), retained intron (RI), mutually exclusive exons (MXEs), alternative 5′ splice site (A5SS), and alternative 3’splice site (A3SS) (31). Differential alternative splicing events were screened using the criteria of |IncLevelDiff| ≥ 0.1, *p*-value ≤0.05, and FDR ≤ 0.05. Key candidate genes related to HF development in sheep were identified via combined analysis of differential alternative splicing events, DETs, and hub genes. The expression, splicing types, and locations of these candidate genes were analyzed in depth. Motifs in the splice site sequences of skipped exons with significant splicing changes were identified using the software XSTREME (v.5.5.5) (32). These splice site sequences replaced the 500 bp downstream sequence of the exon (exonStart-exonEnd: 89851506–89,851,692).

### RT-qPCR

Six DETs (*MT1C*, *KRT27*, *TPRXL*, *FABP9*, *KRT23*, and *S100A14*) were randomly selected, and RT-qPCR was used to determine their relative expression. Exon sequences of the relevant transcripts were downloaded from The National Center for Biotechnology Information (NCBI) database, and primers were designed using Primer Premier 6.0, with primer specificity verified using NCBI Primer-BLAST. GAPDH served as the internal reference gene, with primers synthesized by Bioengineering Co. (Shanghai; Supplementary Table S1). cDNA was synthesized from the extracted total RNA using a PrimeScript™ RT kit (Takara, China). qRT-PCR was performed using a TB Green® Premix Ex Taq™ Fluorescence Quantification kit to determine transcript expression. The qRT-PCR reaction system included the following: 10 μL TB Green Premix Ex Taq II (Tli RNaseH Plus), 0.8 μL forward primer (10 μmol/L), 0.8 μL reverse primer (10 μmol/L), 0.4 μL ROX reference dye (50×), 2 μL cDNA, and 6 μL RNase-free water, made up to a total volume of 20 μL. The real-time fluorescence quantitative PCR procedure was as follows: pre-denaturation at 95°C for 30 s; denaturation at 95°C for 10 s, annealing at 57.8°C for 30 s, and extension at 65°C for 5 s, for a total of 40 cycles. Data were organized using Microsoft Excel (v.2019), and relative transcript expression was calculated using the 2^-ΔΔCt^ method. GraphPad software was used to analyze the graphs.

## Results

### Histological changes in the HFs of sheep

**Figure 1:**
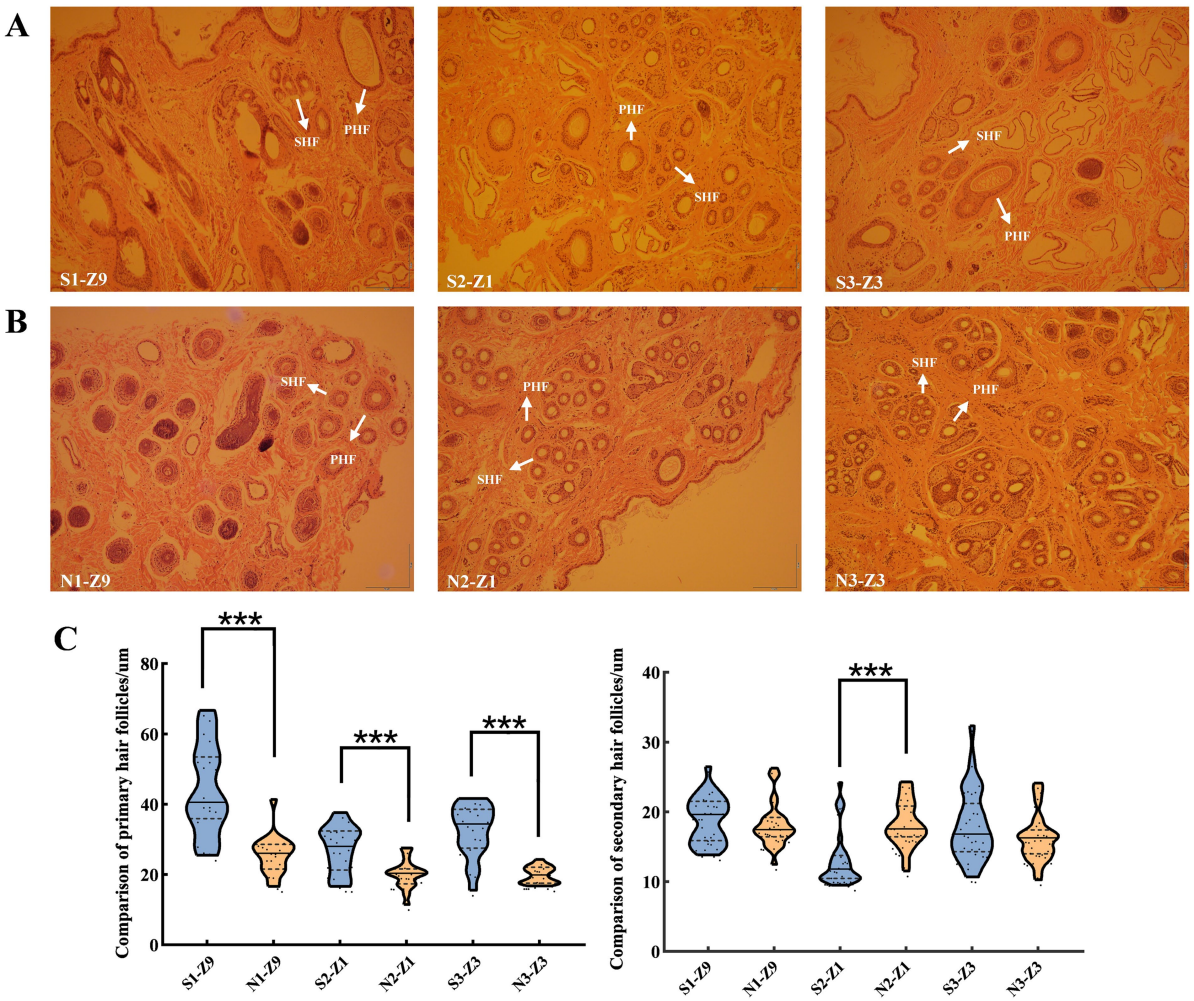
Histological comparison of sheep HFs at 3 developmental stages. (A) and (B) display the histological changes in HFs observed in September, January, and March, respectively (magnification: ×40). (A) September (S1), January (S2), and March (S3). (B) September (N1), January (N2), and March (N3). (C) Measurement of HF diameter (μm) of PHFs and SHFs at 3 developmental stages of sheep HFs and one-way analysis of variance (mean ± standard deviation). 20 PHFs and 30 SHFs were selected for each sample to statistically analyze the mean and standard deviation. In total, the diameters of 120 PHFs and 180 SHFs were measured. Note: HF: hair follicle, PHF: primary hair follicle, and SHF: secondary hair follicle. ***p < 0.001indicates extremely significant difference. Z1, Z3, and Z9 represent the sampling years. Z1, Z3, and Z9 represent the sampling years. Z9: September 2019, Z1: January 2020, Z3: March 2020.

Skin samples were sectioned along the horizontal axis to observe the histological changes in sheep wool follicles during September 2019, January 2020, and March 2020 (Figures 1A,B). In group S, PHFs and SHFs transitioned from the anagen phase to the telogen phase between S1 and S2, with SHFs appearing smaller or even atrophied. Both PHFs and SHFs from S2 to S3 showed signs of developing hair buds and re-entered growth and development. In group N, the PHFs in group N2 still had developing hair buds, and the SHFs did not exhibit significant shrinkage compared with those in group S2. PHFs and SHFs in groups N2 to N3 were in a slow anagen phase. These results indicated that HFs in group S exhibited a rhythmic cyclic development, whereas those in group N lacked a telogen phase, resulting in a continuous growth and development process without a cyclic pattern. Next, we standardized the diameter measurements (μm) of PHFs and SHFs in groups S and N. Highly significant differences were noted between the PHFs in both groups S and N at all 3 time points. However, SHFs were significantly different only in January and not significantly different in either September or March (Figure 1C; Supplementary Table S2). Wool shedding refers to the cycle of growth and development of SHFs, shedding, and regrowth. Therefore, S1, N1, and N2 were initially defined as the anagen phase, S2 as the telogen phase, S3 as the early anagen phase, and N3 as the slow anagen phase. Additionally, the observation of tissue sections provided a reliable basis for selecting samples from specific HF developmental states for transcriptome sequencing and subsequent analysis to identify the transcript related to HF development in sheep.

### Quality assessment results and sample correlation analysis

**Figure 2:**
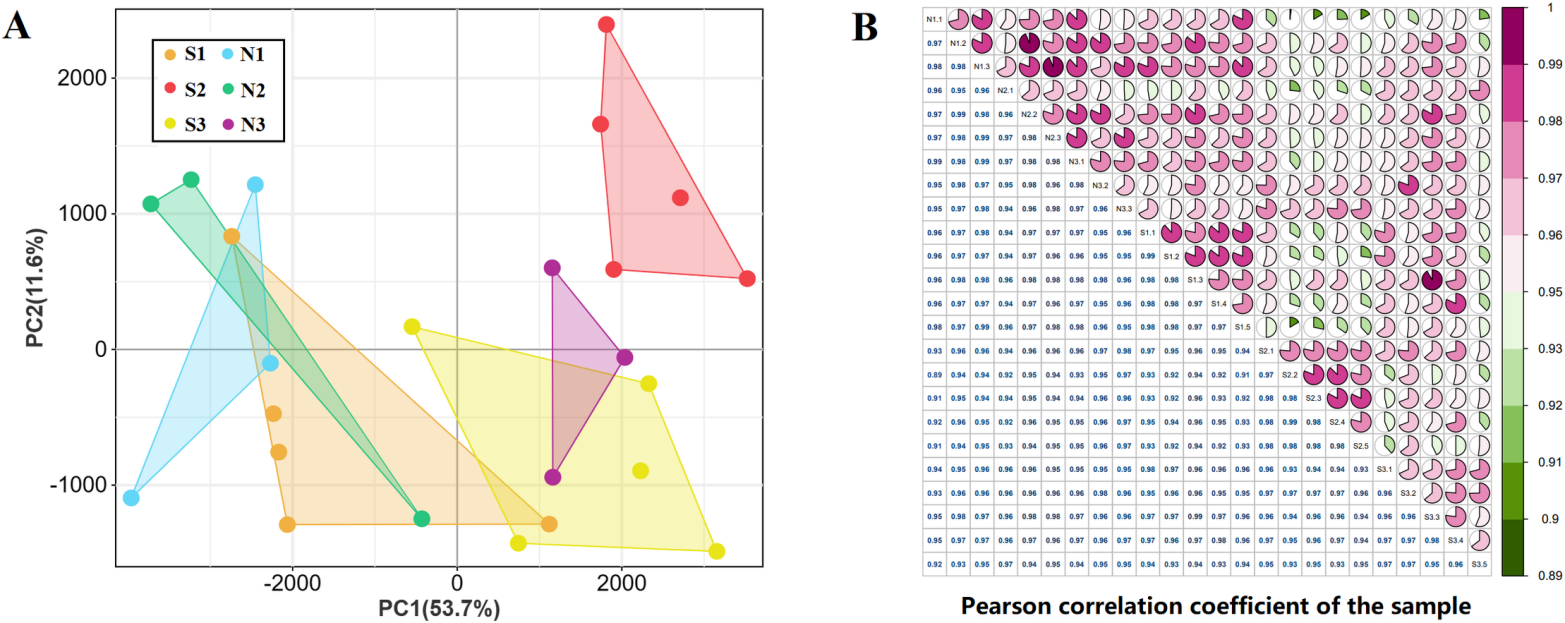
sample correlation analysis. (A) Principal component analysis of 24 samples. (B) Pearson correlation analysis of 24 samples.

To investigate the regulatory mechanisms governing HF growth and development in sheep, a total of 24 samples from both groups S (5) and N (3) were analyzed. The experiment yielded 2,178,092,054 clean reads (Supplementary Table S3) and detected 47,660 transcripts through RNA-seq data analysis (Supplementary Table S4). PCA revealed that S1 and S3 samples were closer to each other, whereas S2 samples were farther from both S1 and S3. By combining the sampling time and section results, it was determined that S1 and S3 were in the anagen phase of HF development, whereas S2 was in the telogen phase. N1 and N2 clustered with S1 and N3 showed expression similar to S3, further suggesting that there was no telogen phase in the HFs of group N sheep. Therefore, it was determined that S1, N1, and N2 were in the anagen phase, S2 was in the telogen phase, S3 was in the early anagen phase, and N3 was in the slow anagen phase (Figure 2A). Correlation analysis was conducted on all samples, and the correlation of duplicate samples within the same treatment group was found to be higher than 0.95 (Figure 2B).

### RT-qPCR validation of RNA-seq

**Figure 3:**
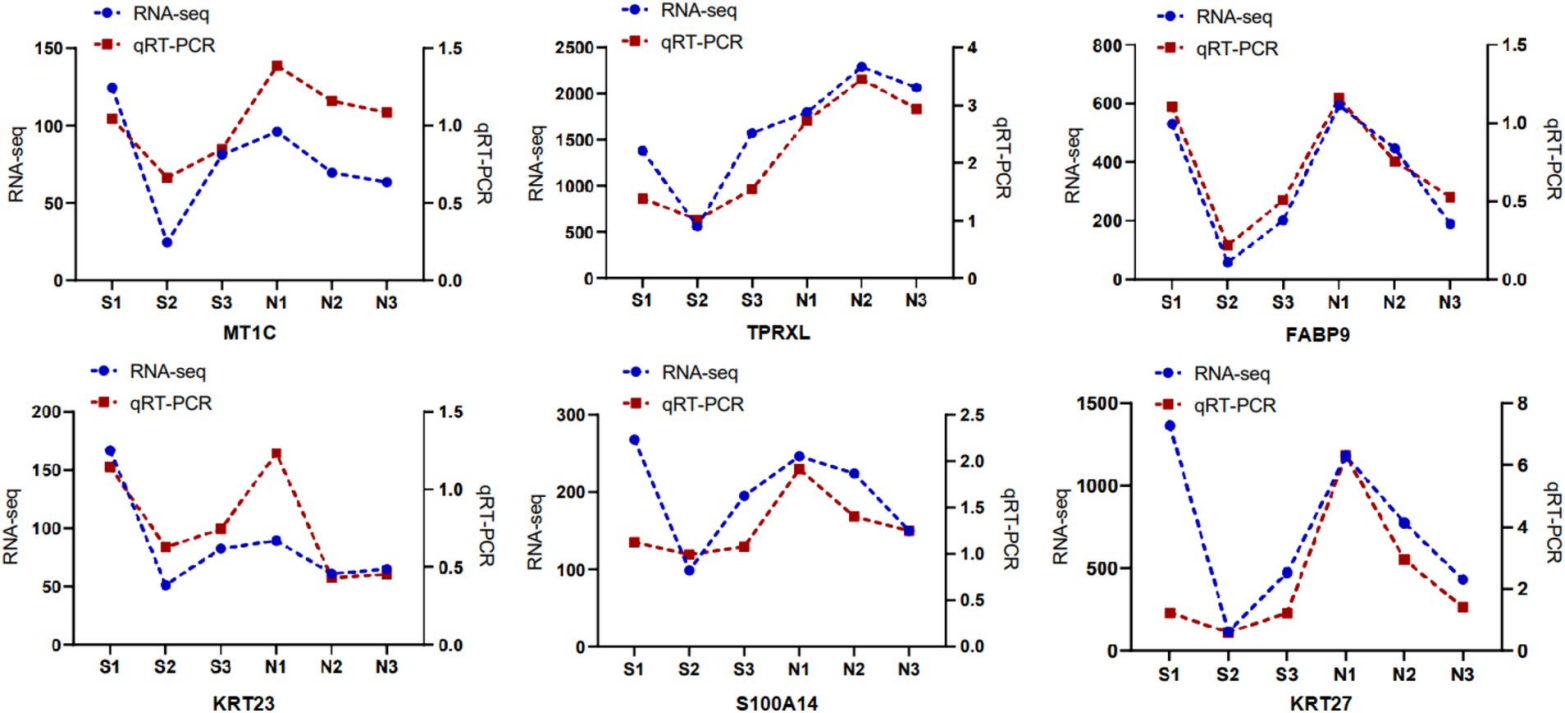
RT-qPCR results.

Six DETs were randomly selected and their comparative expression was determined using RT-qPCR. Results from RT-qPCR for these 6 DETs were consistent with the transcript expression profiles, validating the reliability of the transcript sequencing results (Figure 3).

Identification of differential transcripts and their enrichment analysis.

**Figure 4:**
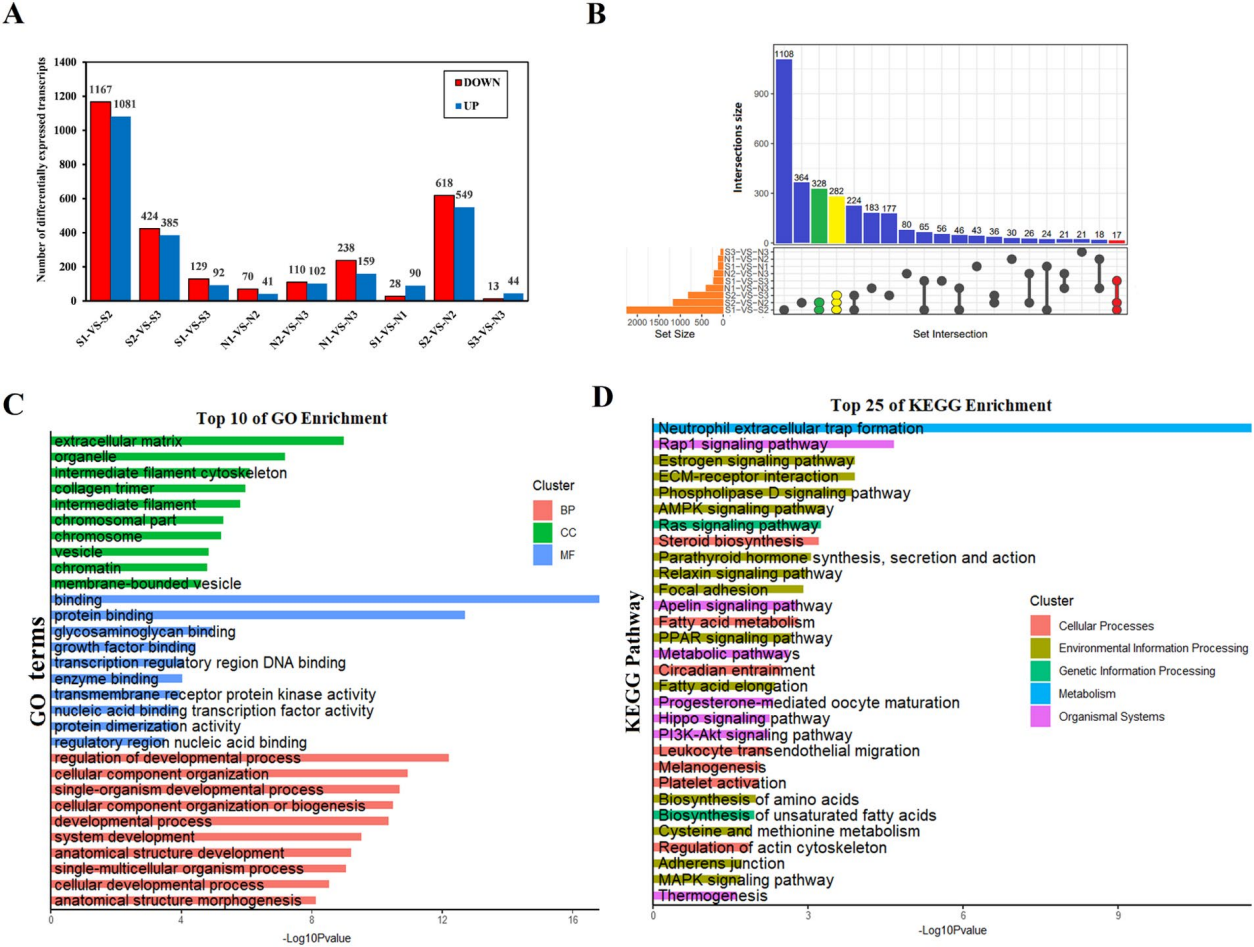
Characteristics of sheep skin tissue at different developmental stages. (A) Relationship between the number of DETs in wool sacs of sheep at different developmental stages. (B) Venn diagram of DETs. Due to the large number of comparison groups, data are presented in the form of an UpSet diagram. (C) Gene Ontology enrichment function results of DETs. biological process (BP), cellular component (CC), and molecular function (MF). (D) Kyoto Encyclopedia of Genes and Genomes enrichment analysis results of DETs (top 25).

A total of 5,340 DETs were identified using the DESeq2 package with |log2FC| ≥ 1 and P-adjust <0.05 as the screening criteria (Figure 4A; Supplementary Table S5). These included 3,278 DETs for intra-Group S comparisons, 720 DETs for intra-Group N comparisons, and 1,342 DETs for comparisons between Groups S and N. Among the 9 groups that were compared, 3 DET sets were of particular interest, all intersecting with N2 and comprising 627 DETs (Figure 4B). GO and KEGG analyses were performed on 5,340 DETs. In the biological process (BP) category, DETs were mainly enriched in the regulation of developmental processes, cellular component organization, and single-organism developmental processes (Figure 4C; Supplementary Table S6). Within the CC category, DETs were predominantly associated with the extracellular matrix, organelles, and the intermediate filament cytoskeleton (Figure 4C; Supplementary Table S6). Within the MF category, DETs were chiefly related to binding, protein binding, and glycosaminoglycan binding (Figure 4C; Supplementary Table S6). DETs were significantly enriched in the pathways of alcoholism, systemic lupus erythematosus, neutrophil extracellular trap formation, Rap1, estrogen, ECM-receptor interaction, phospholipase D, AMPK, and Ras signaling (Figure 4D; Supplementary Table S7).

### Enrichment analysis of hair follicle development-related transcripts

**Figure 5:**
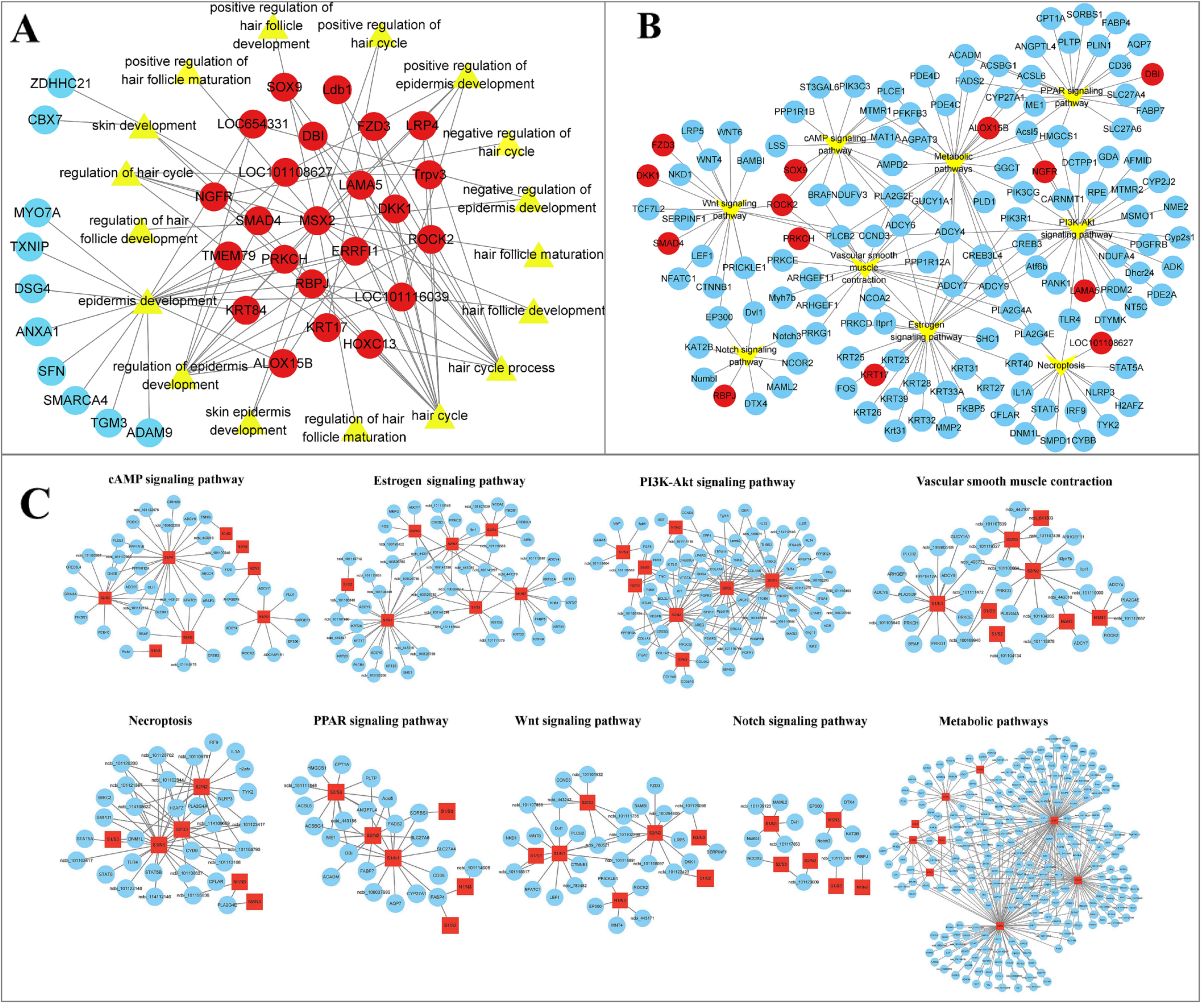
Visualization of GO terms and DETs connected to the development of HFs in sheep. (A) Gene network diagram of biological process (BP) terms during HF morphogenesis in sheep. Red indicates joint effects across multiple BP terms, whereas blue indicates effects in a single BP term. The transcript numbers corresponding to the genes are listed in Supplementary Table S8. (B) Network visualization of Kyoto Encyclopedia of Genes and Genomes (KEGG) pathways and their associated genes during HF morphogenesis in sheep. (C) Occurrence of signaling pathways, including Wnt, Notch, PPAR, estrogen, metabolic pathway, cAMP, PI3K-Akt, necroptosis, and vascular smooth muscle contraction, during sheep wool follicle morphogenesis. GO and KEGG functional enrichment analyses on pathways potentially related to sheep HF growth and development at various phases. The visual network diagram is displayed in Supplementary Figure S1.

To further identify the biological functions associated with HF development in sheep and their potential key transcripts. Cytoscape software was used to visualize GO terms and KEGG pathways in HF development. Enriched BP terms included hair cycle, hair cycle process, regulation of the hair cycle, follicle development, negative regulation of the hair cycle, positive regulation of follicle maturation, positive regulation of HF development, regulation of HF maturation, HF maturation, dermal development, regulation of epidermal development, positive regulation of epidermal development, and negative regulation of epidermal development. Relevant transcripts include the 23 genes, namely, *KRT17*, *ROCK2*, *PRKCH*, *Ldb1*, *RBPJ*, *DBI*, *ERRFI1*, *DKK1*, *LRP4*, *ALOX15B*, *LAMA5*, *Trpv3*, *FZD3*, *MSX2*, *SOX9*, *HOXC13*, *NGFR*, *SMAD4*, *KRT84*, *TMEM79*, *LOC101108627*, *LOC101116039*, and *LOC654331* (Figure 5A; Supplementary Table S8). Similarly, in pathway analyses, 13 genes (*KRT17*, *PRKCH*, *RBPJ*, *DBI*, *DKK1*, *FZD3*, *SMAD4*, *ALOX15B*, *LAMA5*, *NGFR*, *SOX9*, *ROCK2*, and *LOC101108627*) were found to be enriched in Wnt, Notch, PPAR, estrogen, metabolic pathway, cAMP, PI3K-Akt, necroptosis, and vascular smooth muscle contraction signaling pathways. Interestingly, alongside the genes associated with GO terms and KEGG pathways, various genes, including *LEF1*, *CTNNB1*, *WNT6*, *KRT25*, *WNT4*, *KRT40*, *KRT27*, and *BAMBI*, were associated with our traits of interest (Figure 5B). Analogous to the previous GO and KEGG analyses, GO and KEGG analyses were conducted on the pathways potentially related to sheep wool follicle growth and progression at various stages. Additionally, a gene network diagram for these pathways was constructed according to their respective stages (Figure 5C; Supplementary Figure S1). Our results matched those described above and also uncovered several genes in sheep that may be involved in HF growth and development that interact with the above signaling pathways.

### Dynamic expression patterns of DETs during HF development in sheep

**Figure 6:**
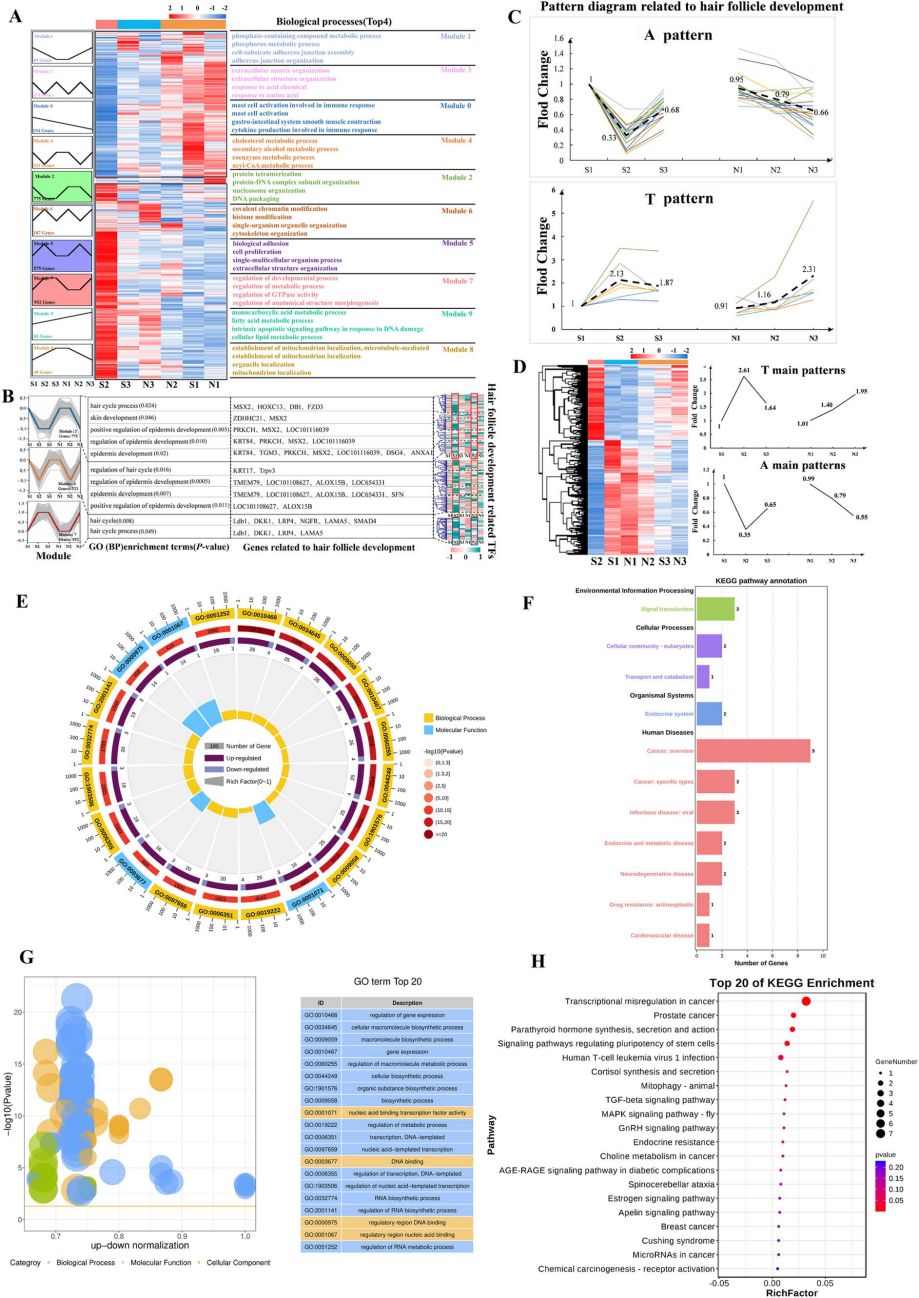
Time-series expression analysis of DETs. (A) Pattern analysis reveals that HFs development in sheep is stage-specific. Heatmap cluster analysis divides DETs into 2 modules, high expression in S2 and low expression in S2 (middle). On the left, DETs are primarily concentrated in 10 expression modules, with the enriched terms related to the corresponding biological processes (Top 4) shown on the right. (B) Genes related to HF development in sheep are primarily concentrated in modules 2, 4, and 7 (*p* < 0.05). The corresponding biological processes, genes, and TFs are shown on the right. The transcript numbers corresponding to the genes are listed in Supplementary Table 10. (C) Patterns A (intense expression throughout the anagen phase) and T (intense expression during the telogen phase). Bold dashed lines indicate the main modes (average) of A and T patterns. (D) Summary of the A and T pattern of all DETs. (E–G) TFs are mainly enriched in Gene Ontology terms (Top 20). Visualization using enrichment circles and z-score bubble charts. (F–H) TFs are mainly enriched in Kyoto Encyclopedia of Genes and Genomes terms (Top 20). Bubble charts and secondary classification bar charts are shown.

A hierarchical cluster analysis of all DETs was performed to determine the expression patterns of DETs associated with HF development in sheep. This analysis revealed 2 major expression modules, namely, high and low expression during the S2 period (Figure 6A). Next, a time-series expression analysis was conducted on all DETs, which were classified into 10 expression modules based on changes in their expression patterns (Figure 6A). GO functional enrichment analysis was performed separately on these 10 modules, which identified 11 BP terms related to HF development in sheep (Figure 6A; Supplementary Table S9). In Module 2, transcripts associated with HF development were enriched for processes including hair cycle, skin development, positive regulation of epidermis development, regulation of epidermis development, and epidermis development. These transcripts were characterized by low expression at S2 and high expression at N2 (Figure 6B; Supplementary Table S10). Module 4 was associated with similar processes, including regulation of hair cycle, regulation of epidermis development, epidermis development, and positive regulation of epidermis development, with the transcripts also showing low expression at S2 (Figure 6B; Supplementary Table S10). Analysis of the transcripts related to HF development in Modules 2 and 4 revealed that most showed strong expression in the anagen phase (S1), low expression during the telogen phase (S2), and a slight increase in expression during the early anagen phase (S3). In contrast, the N group exhibited a consistent decrease in expression (N1 and N2 in the anagen phase, and N3 in the slow anagen phase), which was defined as the A pattern (Figure 6C). Module 7 plays a key role in the hair cycle and hair cycle processes (Figure 6B; Supplementary Table S10). Its expression pattern is the opposite of that observed in Modules 2 and 4 (low expression at S1, high expression at S2, and slightly reduced expression at S3). In the N group, the expression of N1, N2, and N3 increased consistently, and this was defined as the T pattern (Figure 6C).

Motif enrichment analysis was performed on the promoters (2000 bp upstream and 500 bp downstream of the transcription start site) of HF development-related transcripts in Modules 2, 4, and 7 to determine whether the stage-specifically expressed transcripts were co-regulated by certain TFs. Motifs for TFs were predominantly enriched in the Kruppel-like factor family, Sp family, and ZNF family (Supplementary Table S11). Analysis of the expression patterns of these TFs revealed significant differences between S2 and N2, suggesting that these TFs may play an important role in regulating normal HF growth cycles (Figure 6B). TFs showed significant enrichment in BPs, including the regulation of gene expression, cellular macromolecule biosynthetic process, macromolecule biosynthetic process, and gene expression (Figures 6E–G). They are also involved in the dynamic regulation of signaling pathways including TGF-*β*, GnRH, Estrogen, and Apelin pathways (Figures 6F–H). Additionally, for screening A and T pattern transcripts among all DETs, a total of 1,005 A and T pattern transcripts were identified, comprising 605 A pattern and 400 T pattern transcripts (Figure 6D; Supplementary Table S12). KEGG analysis revealed that A pattern transcripts were primarily enriched in pathways related to systemic lupus erythematosus, alcoholism, neutrophil extracellular trap formation, estrogen signaling, necroptosis, and metabolic pathways (Figure 6D; Supplementary Figures S2A,B), whereas T pattern transcripts were mainly enriched in pathways associated with salivary secretion, oxytocin signaling, Apelin signaling, circadian entrainment, cGMP-PKG signaling, and Rap1 signaling (Figure 6D; Supplementary Figures S2C,D). The clustering of A and T pattern transcripts within these signaling pathways suggests a close relationship between A and T pattern transcripts and HF development.

### WGCNA analysis identifies stage-specific co-expression modules

**Figure 7:**
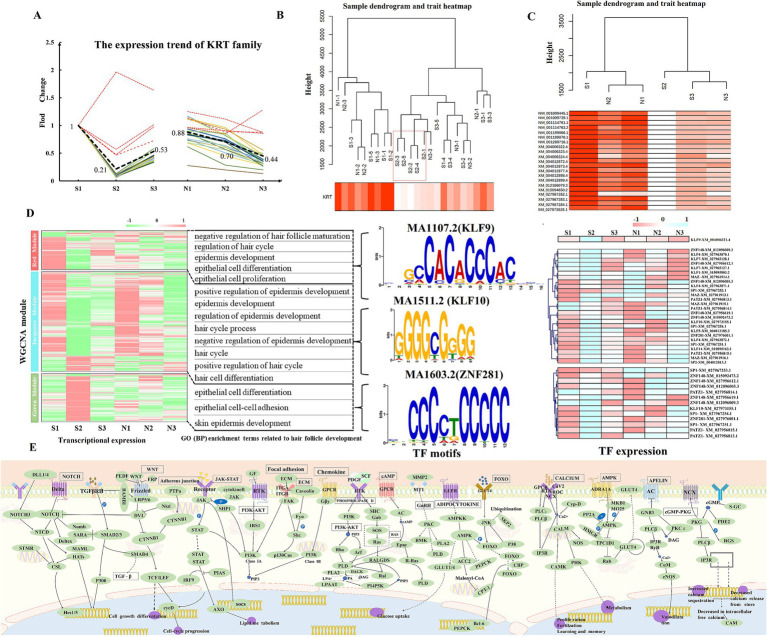
Weighted gene co-expression network analysis. Note: (A) Normalized expression pattern of the KRT family. The color indicates the expression pattern of 21 KRTs (A pattern). The bold, black dashed line indicates the main pattern of KRTs, whereas the red dashed line represents the deleted value, which does not appear in the subsequent analysis. (B,C) Hierarchical clustering tree of KRTs and sheep skin transcript expression. Figure B shows the clustering of the main pattern of KRTs and 24 samples, and Figure C shows the clustering of 21 KRTs and transcripts at each stage. (D) Expression clustering of transcripts in 3 key modules. Biological process terms related to HF development corresponding to the key modules are enriched on the right, with related TF genes and leading sequence motifs displayed beside each module. (E) Pathways related to HF development in sheep.

The KRT Family Members are one of the main components of wool tissue structure and serve as an important marker for HF growth and development (17). The expression of 26 KRTs in the sequencing results was analyzed in this study. The overall trend of KRTs is shown as A pattern. The homogenization of the expression of 21 KRTs matching this pattern was calculated (Figure 7A; Supplementary Table S13). Correlation analysis between KRT family as an indicator of hair follicle development status (Phenotypic data) and transcript expression. The results showed that groups S1, N1, and N2 had the highest correlation with the development status data, followed by groups S3 and N3, whereas group S2 showed the least correlation (Figures 7B,C). In this clustering, N2 was grouped with S1 and N1, indicating its closer association with the HF anagen period. The expression of KRTs in the S2 stage was significantly lower than that in the S3 stage (the early anagen phase), being only 24% of the anagen phase level, suggesting that S2 represents the telogen phase of HFs (Figure 7A). In summary, the developmental status of HFs in groups S and N was further confirmed, wherein S1, N1 and N2 corresponded to the anagen phase, S2 corresponded to the telogen phase, S3 cor-responded to the early anagen phase, and N3 corresponded to the slow anagen phase. These results correspond with those from PCA and time-series expression analysis.

To construct the co-expression network of 24 samples, we chose *β* = 5 to construct the TOM clustering tree and merged similar modules (modules with 75% feature gene similarity), resulting in 9 modules (Supplementary Figure S3). The expression pattern heatmap showed that the MEturquoise (cor = 0.95, *p* = 4e-12), MEred (cor = 0.81, *p* = 1e-06), and MEgreen (cor = −0.56, *p* = 0.004) modules were significantly correlated with KRTs (Figure 7D). Therefore, transcripts from these 3 modules were chosen for GO (BP) and KEGG analyses. The red module was significantly enriched for negative regulation of HF maturation, regulation of the hair cycle, negative regulation of the hair cycle, and epidermis development. The green module was enriched for epithelial cell differentiation, epithelial cell–cell adhesion, and skin epidermis development related to HF development. The turquoise module was mainly enriched for positive regulation of epidermis development, positive regulation of HF development, and regulation of epidermis development (Figure 7D; Supplementary Table S14). In combination with DETs and time-series expression analysis, the gene relationships between Wnt, Ras, PI3K-Akt, MAPK, focal adhesion, and Rap1 pathways using KEGG are shown in Figure 7E (Supplementary Table S14). Grounded in the candidate genes connected to HF development in sheep (Supplementary Table S15), 11 core genes (|MM| ≥ 0.75), namely, *DBI*, *FZD3*, *KRT17*, *ZDHHC21*, *TGM3*, *DSG4*, *TMEM79*, *KRT84*, *HOXC13*, *LOC101116039*, and *MSX2* were identified in the red, green, and turquoise modules as being involved in the control of HF growth and development in sheep.

### Analysis of alternative splicing events related to HF development in sheep

**Figure 8:**
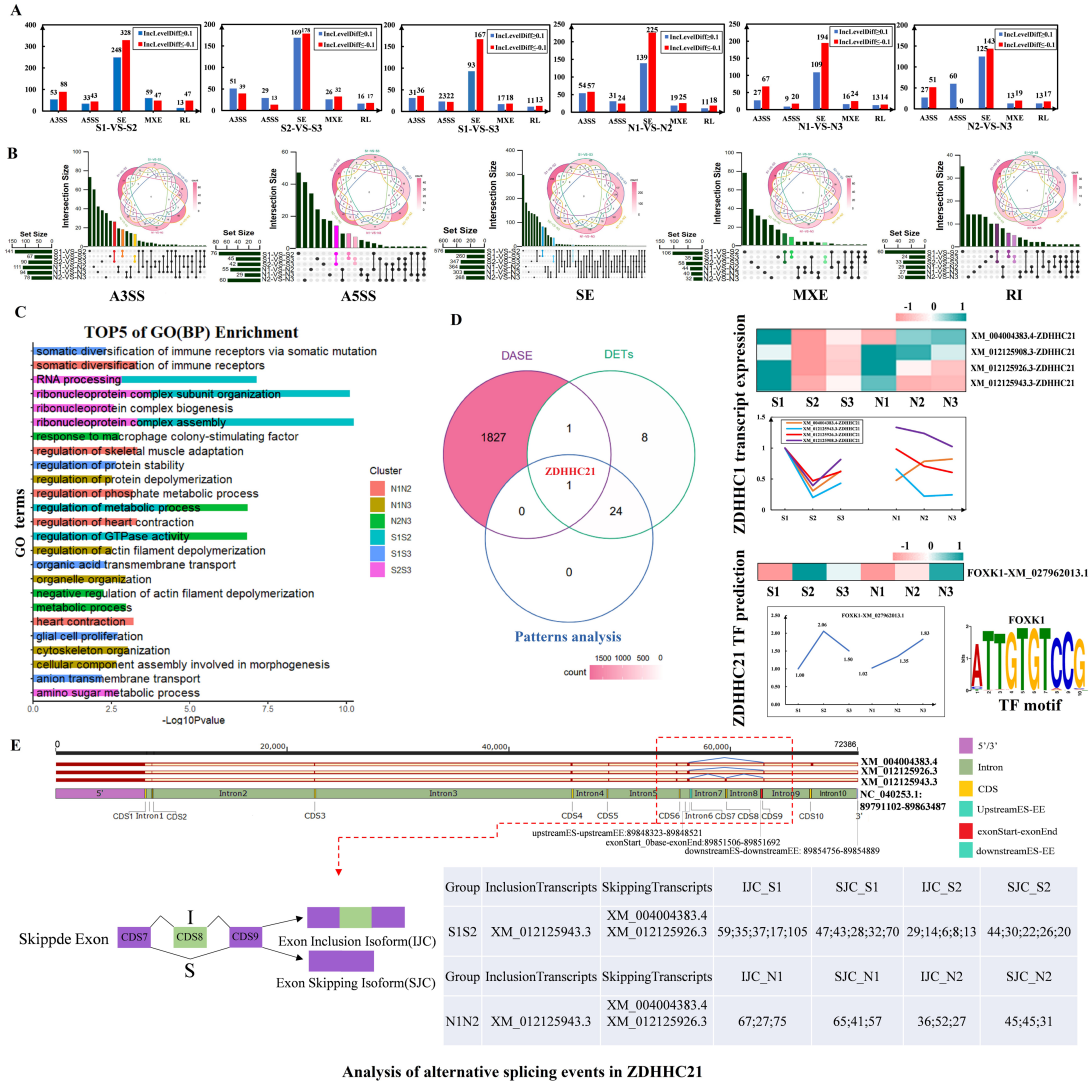
Analysis of alternative splicing events related to hair follicle development in sheep. (A) Analysis of differential alternative splicing events across 3 transition periods. (B) Venn diagram showing the 6 comparison groups. Alternative splicing events that are significantly different only in the S group transition period are highlighted. (C) Top 5 biological processes of the 6 comparison groups. (D) Venn diagram of differential alternative splicing events, differentially expressed transcripts and Pattern Analysis. The right side shows the expression of *ZDHHC21* transcripts, related TFs, and the top representative sequence motifs. (E) Detailed analysis of alternative splicing positions of the *ZDHHC21* gene. IJC indicates the number of relevant reads that support the retention of the skipped exon (exon inclusion), which will appear in the mature mRNA after splicing. SJC records the number of relevant reads supporting the splicing of the skipped exon (exon skipping), which is excised during alternative splicing and does not appear in mature mRNA.

Alternative splicing is an important regulatory modality affecting HF development in sheep. To investigate its effect on the cyclic development of sheep HF, rMTAS software was used to analyze transcriptome data of sheep skin. Differential alternative splicing events were identified in the three transition phases (anagen-telogen, telogen-early anagen, early anagen-anagen) within the S group and the N group. All 6 comparison groups showed the most ES-type differential alternative splicing events (Figure 8A; Supplementary Table S16). Among these, the alternative splicing events that were significantly different only in the transition periods of group S were of particular interest as they may be closely related to HF development in sheep (Figure 8B). GO (BP) enrichment function analysis of the 6 comparison groups showed that they were mainly significantly enriched in metabolic process regulation, negative regulation of actin filament depolymerization, and organization of ribonucleoprotein complex subunits. In addition, they were also significantly enriched in skin development and HF development (Figure 8C). Intersecting these results with the genes related to HF development identified the *ZDHHC21* (Figure 8D). Among its transcripts, XM_012125908.3, XM_004004383.4, XM_012125943.3, and XM_012125926.3 were consistently expressed in group S but abnormally expressed in group N (Figure 8D).

The prediction of transcription factors (TFs) for the *ZDHHC21* revealed that *FOXK1* (XM_027962013.1) exhibited an expression pattern opposite to that of the *ZDHHC21* transcript (Figure 8D). The alternative splicing events in *ZDHHC21* were further analyzed. XM_004004383.4 and XM_012125926.3 of *ZDHHC21* exhibited exon skipping events at CDS8 (89854756–89,854,889), whereas XM_012125943.3 had an intron retention event at CDS8 (Figure 8E). In the S1-*vs*-S2 comparison, the SJC_S2 of *ZDHHC21* was nearly double that of IJC_S2. In contrast, in the N1-*vs*-N2 comparison, the difference between SJC_N2 and IJC_N2 was smaller, highlighting a significant contrast with the S1-*vs*-S2 comparison (Figure 8E). Combining the expression of the 3 transcripts, it was predicted that XM_004004383.4 and XM_012125926.3 of *ZDHHC21* might play a decisive role in HF development in sheep (Figures 8D,E). In summary, there were notable differences in the reads of the final processed mRNA transcripts of *ZDHHC21* after alternative splicing in groups S and N, which may directly affect the growth and developmental status of HFs.

## Discussion

In mammals, each mature HF functions as a regenerative system, enabling animal hair to grow in a regular pattern (33). Understanding the morphological and molecular mechanisms underlying the normal growth and development of wool follicles in sheep is crucial for advancing our knowledge of hair growth biology and the genetic underpinnings of wool traits. In this analysis, we selected skin tissues from Dorper sheep in September, January, and March for histomorphological analysis between the shedding and non-shedding groups. In group S, HFs developed in a rhythmic cyclical manner, from anagen (S1) to telogen (S2) and re-entered the early anagen phase (S3). In group N, HFs in the telogen phase (N2) still exhibited growing and developing hair buds, indicating that the follicles did not follow a cyclic pattern and remained in a constant state of growth and development. Wool shedding was found to be a cyclic process involving the growth and development of SHFs, shedding of wool, and subsequent hair regrowth (4, 5, 34). Standardized diameter measurements (μm) of SHFs in groups S and N revealed highly significant differences in SHFs only in January (telogen phase), with no significant differences observed in September or March. This finding indicated that HFs in the N2 period were in a constant state of growth and development. Additionally, the growth and developmental state of HFs in group S was determined to be in the anagen phase in September, the telogen phase in January, and the early anagen phase in March, which was consistent with that in previous studies (12, 35–37).

From a transcriptome perspective, 3,278, 720, and 1,342 DETs were identified in the S, N, and S-*vs*-N groups. DETs were significantly enriched in signaling pathways such as the Rap1, estrogen, ECM-receptor interaction, phospholipase D, AMPK, NOTCH, MAPK, and Ras pathways. Previous studies have shown that the PI3K-Akt, MAPK, NOD-like receptor, ECM-receptor interaction and Rap1 pathways are involved in the growth and development of hair follicles in cashmere goats (14, 18). Additionally, the PPAR pathway plays a role in HF development, control of keratinocyte differentiation, and the development of functional skin barrier (38, 39). The MAPK signaling pathway is crucial in mammalian HFs and plays a role in keratinocyte differentiation, epidermal cell differentiation, and multicellular biological development (8, 40). High-throughput transcriptome sequencing identified differences in gene expression between primary and secondary hair follicles and showed that angiogenesis, ECM receptor interactions, and the Wnt/*β*-catenin/Lef1 signaling pathway are closely associated with hair follicle morphogenesis (34). The Notch pathway plays a key role in determining cell fate, regulating the proliferation and differentiation of epidermal tissue cells, and wound healing (41, 42). Notch signaling plays a role in the late stage of embryonic hair follicle formation, and hair lacking Notch signaling will appear abnormal (43). In addition, during the development of hair follicles in mice, knocking out β-catenin prevents the formation of dermal condensates, underscoring the critical role of the LEF/TCF/β-catenin signaling pathway in regulating dermal condensate formation (44–47). DKK1 inhibits the Wnt signaling pathway by blocking the phosphorylation of β-catenin, thereby inducing hair follicle regression (48). The PI3K/Akt signaling pathway is essential for maintaining and restoring the hair-inductive capacity of human dermal cells and promoting hair follicle regeneration (49, 50). Furthermore, the PI3K/Akt, Wnt, Notch, and BMP signaling pathways participate in the growth and development of hair follicles and skin tissues in Rex rabbits. These pathways also facilitate the transition of hair follicles from the anagen phase to the catagen and telogen phases, affecting hair density in Rex rabbits (51). In this study, the Rap1, Ras, MAPK, phospholipase D, and estrogen signaling pathways were significantly enriched in the S1-*vs*-S2 comparison group, suggesting that these pathways and their associated genes may regulate the transition of HFs from the anagen to the telogen phase. In the S2-*vs*-S3 comparison group, the PPAR, ECM-receptor interaction, PI3K-Akt, AMPK, and focal adhesion signaling pathways were significantly enriched, indicating that genes in these pathways may play a role in the transition from the telogen to the early anagen phase, stimulating the generation of new hair shafts and leading to the timely shedding of old wool. The Rap1, estrogen, and Ras pathways were significantly re-enriched in the S3-*vs*-S1 comparison group, suggesting their crucial role in facilitating the cycling process of HFs from the early anagen phase to the anagen phase. The N1-*vs*-N2 comparison group did not show significant enrichment in pathways such as Rap1, estrogen, MAPK, and phospholipase D pathways compared with that in the S group. However, these pathways appeared in the N2-*vs*-N3 comparison group. The Rap1, estrogen, ECM-receptor interaction, and Ras pathways were significantly enriched in the N3-*vs*-N1 comparison group and were similar to the enrichment results noted in the S3-*vs*-S1 comparison group. These results indicated that the HFs in group N did not enter the telogen phase. Thus, it could be concluded that the HFs in group N did not follow a cyclic pattern and were always in a state of growth and development. The results of this study align with findings from previous research on cashmere goats, mice, and even human dermal cells. The PI3K-Akt signaling pathway and ECM-receptor interactions play important roles in the transition from the telogen to the anagen phase and serve as candidate biomarkers for this regeneration (14). Compared to our study, the PI3K/Akt pathway in Rex rabbits primarily facilitates the transition of hair follicles from the anagen phase to the telogen, contrary to our findings, suggesting that PI3K/Akt expression may vary across different mammals. Furthermore, The PI3K-Akt signaling pathway is crucial for regulating keratinocyte survival and proliferation, among other functions (52).

Using time series expression analysis and WGCNA, *MSX2* (53), *HOXC13* (54), *DBI*, *FZD3* (55), *ZDHHC21* (56), *PRKCH*, *LOC101116039*, *KRT17* (57), *DSG4* (58), *TMEM79* (59), and *LOC101108627* were determined to be associated with sheep hair follicle development. Additionally, *ALOX15B*, *LOC654331*, *SFN*, *Ldb1* (60), *DKK1* (61), *NGFR* (62), *LAMA5* (63), *SMAD4* (64), and *LRP4* (65, 66) were found to be associated with hair cycle processes, skin development, epidermis development, and hair cycle regulation. The above genes related to hair follicle development showed stage-specific expression, with *MSX2*, *HOXC13*, *KRT17, DBI*, *FZD3*, and *ZDHHC21* showing the highest expression during the anagen phase (A pattern). In contrast, *Ldb1*, *DKK1*, *NGFR*, *LAMA5*, *SMAD4*, and *LRP4* were highest expressed during the telogen phase (T pattern). In addition, The KRT family is a major component of wool, and the keratins *KRT25*, *KRT27*, *KRT19*, *KRT10*, *KRT77*, *KRT1*, and others are regarded as markers of the hair follicle cycle. They are significantly enriched in estrogen and ECM-receptor interaction signaling pathways and play a very important role in the development of secondary hair follicles (17). KRTs have been associated with the development of the hair follicle in yaks, and gene expression correlation analysis of the keratin family revealed a strong positive correlation of KRTs mainly throughout the hair follicle development cycle (67). TFs such as *TCF3*, *TCF4*, *ZNF740*, *EGR1*, *FLI1*, *SP1*, *E2F6*, and *ZNF148*, among others, were predicted to regulate these genes associated with HF growth and development. Signaling pathways were significantly enriched with these TFs, as revealed by functional enrichment analysis such as the TGF-*β*, GnRH, estrogen, and apelin pathways. Previous studies have demonstrated that transcription factors such as *EGR1*, *LEF1*, *HOXC13*, *RBPJ*, *VDR*, *RARA*, and *STAT3* have stage-specific roles in HF morphogenesis (11). The study shows that *EGR1* is closely associated with HF growth and development and plays a crucial role in embryonic organ formation (68–71). The TFs Lefl and Twisare expressed in the early stages of dermal condensates (44, 47). The role of *CUX1 in vitro* dermal papilla cell proliferation may be regulated by *SP1* and *KROX20* (72). Additionally, *ZDHHC21* has been identified as the key gene affecting HF morphogenesis in Merino sheep (18). Members of the ZDHHC family mediate post-translational modifications via palmitoylation, with the *ZDHHC21* protein being specifically expressed in the skin and restricted to a particular hair lineage. Loss of *ZDHHC21* function results in delayed hair shaft differentiation at the gene expression level (56). In the current study, analysis of alternative splicing events in the *ZDHHC21* gene revealed a significant exon skipping event in the S1-*vs*-S2 comparison, with SJC_S2 being almost twice that of IJC_S2, unlike that in the N1-*vs*-N2 comparison. Based on the transcript expression of the *ZDHHC21* gene, we could predict that the isoforms XM_004004383.4 and XM_012125926.3 may play a crucial role in HF development in sheep. Additionally, alternative splicing, a key mode of transcriptional regulation, showed differences in the mRNA reads of the *ZDHHC21* Isomers between groups S and N, which may directly influence HF growth and development.

## Conclusion

RNA-seq was used to sequence skin tissues from sheep HFs during the anagen, telogen, and early anagen phases, which led to the identification of key candidate genes and their corresponding transcripts associated with HF development in sheep. For example, *HOXC13*, *DBI*, *FZD3*, *ZDHHC21*, *KRT8*, and *SMAD4*. Several signaling pathways related to HF development, including the Rap1, Ras, MAPK, Jak–STAT, and PI3K-Akt pathways, were identified across the 3 HF development stages. Furthermore, it was found that the XM_004004383.4 and XM_012125926.3 transcripts of the *ZDHHC21* gene may play a decisive role in the development of HF in sheep. This study serves as a valuable resource for interpreting the morphology of normal HF growth and development in sheep, elucidating the genetic basis of mammalian skin–associated traits, and offering potential insights into the molecular mechanisms of human hair growth and development.

## Data Availability

The datasets presented in this study can be found in online repositories. The names of the repository/repositories and accession number(s) can be found at: https://www.ncbi.nlm.nih.gov/bioproject; PRJNA963059.

## Footnotes

^1^ https://imagej.net/ij/download.html

^2^ https://daehwankimlab.github.io/hisat2/

^3^ www.omicshare.com/tool
