# Supplementary material for: Unlocking the genetic secrets of Dorper sheep: insights into wool shedding and hair follicle development
Source: Front Vet Sci. 2024 Dec 11;11:1489379. doi: 10.3389/fvets.2024.1489379 (PMC11670804; doi:10.3389/fvets.2024.1489379)
Supplement: Supplementary file 2 [file Supplementary_file_1.zip › Appendix Figure/Appendix Figure.docx]

**Figure S1** Visualization of Genes and Pathways Associated with Sheep Wool Follicle Development in Comparison Groups.





**Figure S2** KEGG Enrichment Analysis of A and T Pattern Transcripts Among All DETs.


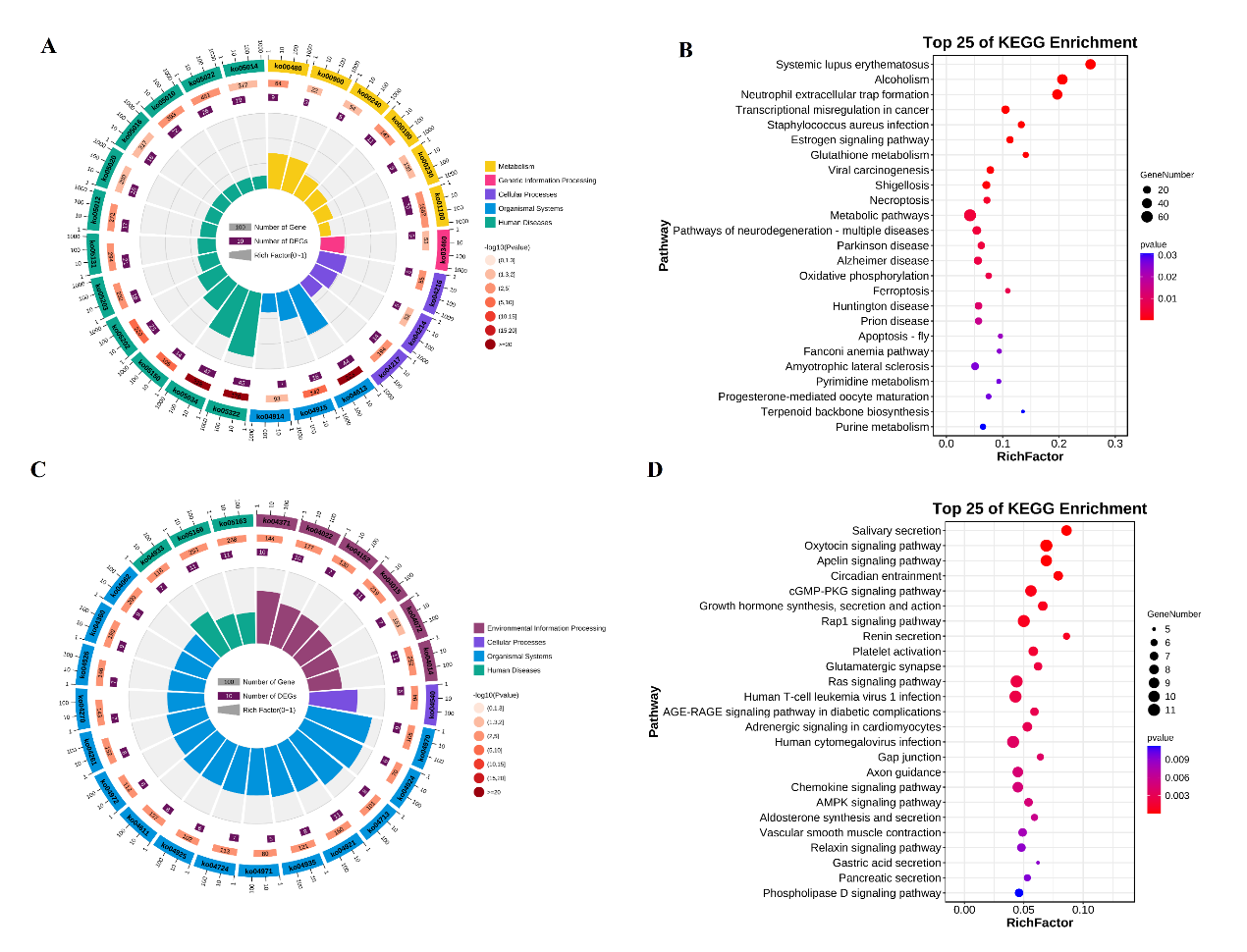


**Figure S3** Weighted Gene Co-Expression Network Analysis for Identifying Co-Expression Modules.


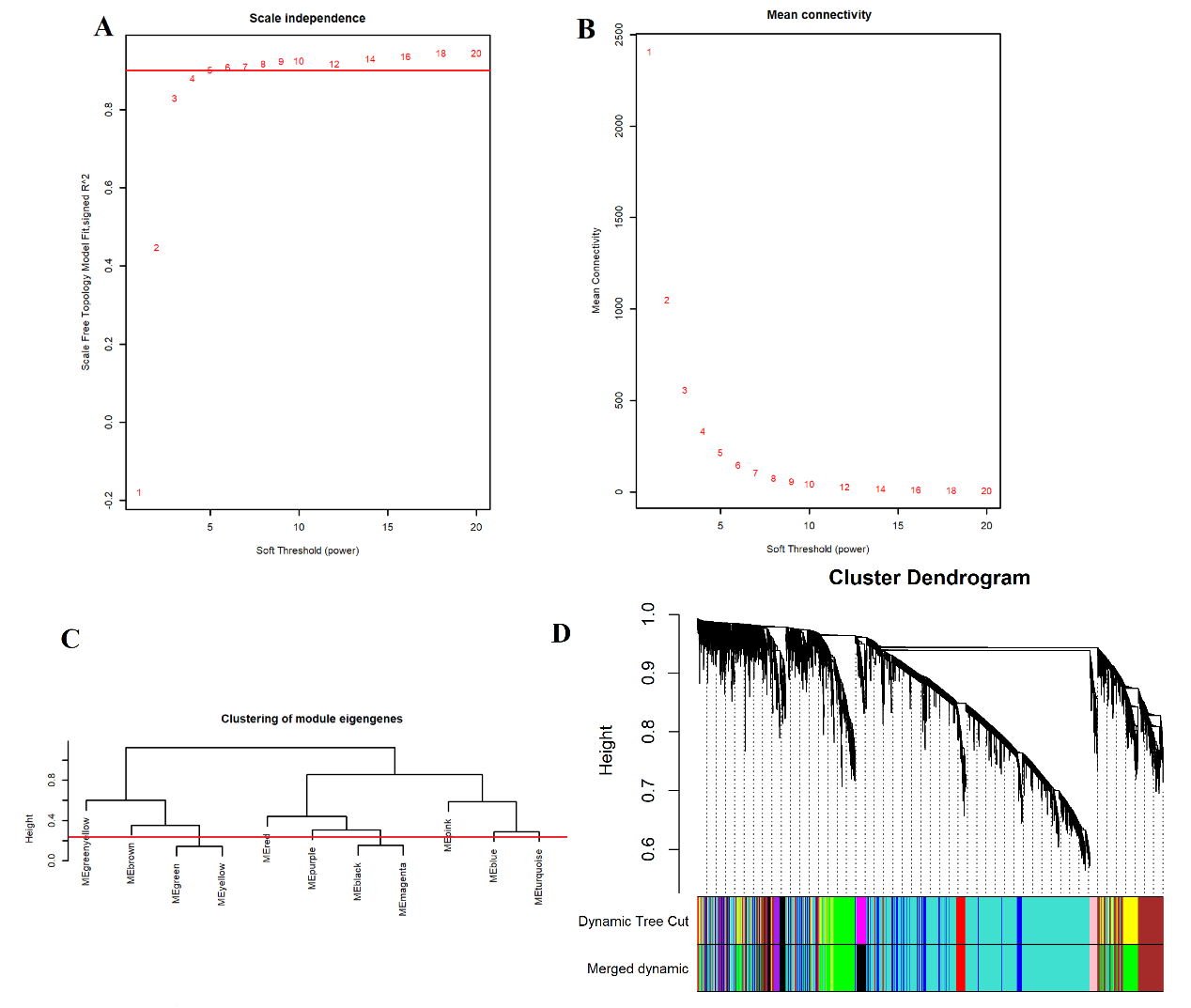


Note: (A) and (B) Soft Threshold Power Determination; (C) Cluster Dendrogram of Characteristic Genes in Gene Modules. (D) The gene clustering dendrogram was obtained according to neighbor-joining based non-dissimilarity hierarchical clustering.
